# Yerba mate (*Ilex paraguariensis,* A. St.-Hil.) de novo transcriptome assembly based on tissue specific genomic expression profiles

**DOI:** 10.1186/s12864-018-5240-6

**Published:** 2018-12-07

**Authors:** Jessica V. Fay, Christopher J. Watkins, Ram K. Shrestha, Sergio L. Litwiñiuk, Liliana N. Talavera Stefani, Cristian A. Rojas, Carina F. Argüelles, Julian A. Ferreras, Mario Caccamo, Marcos M. Miretti

**Affiliations:** 1 Grupo de Investigación en Genética Aplicada (GIGA), Facultad de Ciencias Exactas Químicas y Naturales, Instituto de Biología Subtropical (IBS UNaM-CONICET), Universidad Nacional de Misiones, Jujuy 1745, CP3300 Posadas, Misiones Argentina; 2 The Genome Analysis Centre, Norwich Research Park, Norwich, NR4 7UH UK; 3 Present address: Earlham Institute, Norwich Research Park, Norwich, NR4 7UZ UK; 4 Present address: NIAB, Huntingdon Road, Cambridge, CB3 0LE UK; 5 Universidad Federal de la Integración Latinoamericana, Foz de Iguazú, PR Brazil

**Keywords:** *Ilex paraguariensis*, *Yerba mate*, Transcriptome, Expression profile, Functional annotation, de-novo assembly, Phenylpropanoid

## Abstract

**Background:**

The most common infusion in southern Latin-American countries is prepared with dried leaves of *Ilex paraguariensis* A. St.-Hil., an aboriginal ancestral beverage known for its high polyphenols concentration currently consumed in > 90% of homes in Argentina, in Paraguay and Uruguay. The economy of entire provinces heavily relies on the production, collection and manufacture of *Ilex paraguariensis*, the fifth plant species with highest antioxidant activity. Polyphenols are associated to relevant health benefits including strong antioxidant properties. Despite its regional relevance and potential biotechnological applications, little is known about functional genomics and genetics underlying phenotypic variation of relevant traits. By generating tissue specific transcriptomic profiles, we aimed to comprehensively annotate genes in the *Ilex paraguariensis* phenylpropanoid pathway and to evaluate differential expression profiles.

**Results:**

In this study we generated a reliable transcriptome assembly based on a collection of 15 RNA-Seq libraries from different tissues of *Ilex paraguariensis*. A total of 554 million RNA-Seq reads were assembled into 193,897 transcripts, where 24,612 annotated full-length transcripts had complete ORF. We assessed the transcriptome assembly quality, completeness and accuracy using BUSCO and TransRate; consistency was also evaluated by experimentally validating 11 predicted genes by PCR and sequencing. Functional annotation against KEGG Pathway database identified 1395 unigenes involved in biosynthesis of secondary metabolites, 531 annotated transcripts corresponded to the phenylpropanoid pathway. The top 30 differentially expressed genes among tissue revealed genes involved in photosynthesis and stress response. These significant differences were then validated by qRT-PCR.

**Conclusions:**

Our study is the first to provide data from whole genome gene expression profiles in different *Ilex paraguariensis* tissues, experimentally validating *in-silico* predicted genes key to the phenylpropanoid (antioxidant) pathway. Our results provide essential genomic data of potential use in breeding programs for polyphenol content. Further studies are necessary to assess if the observed expression variation in the phenylpropanoid pathway annotated genes is related to variations in leaves’ polyphenol content at the population scale. These results set the current reference for *Ilex paraguariensis* genomic studies and provide a substantial contribution to research and biotechnological applications of phenylpropanoid secondary metabolites.

**Electronic supplementary material:**

The online version of this article (10.1186/s12864-018-5240-6) contains supplementary material, which is available to authorized users.

## Background

*Ilex paraguariensis* A. St.-Hil (*Aquifoliaceae),* known as “Yerba Mate” (YM), is a South American native tree species widely cultivated in North-East Argentina, South-West Brazil and Eastern Paraguay [1]. Argentina has the largest cultivated area (152,000 ha), with 87% located in a single province: Misiones [2].

For centuries the “mate”, a beverage made from an infusion of YM dried leaves, has been widely consumed in South America countries (southern Brazil, Argentina, Paraguay, and Uruguay). Recent surveys showed that “mate” is consumed in 98% of the Argentinean homes http://yerbamateargentina.org.ar/ and it is globally increasing in popularity due to its health benefits [3]. A number of research reports confirmed relevant pharmacological properties, such as antioxidant activity [4], protective effects against induced DNA damage and atherosclerosis [5, 6], potent anti-obesity activity and modulatory effect on glucose levels [7]. These properties transform “mate” into a conceivable functional food with active principles with a relevant biochemical profile for the pharmaceutical industry. In addition, “mate” is a potential carrier food for functional ingredients or supplements with extensive spread consumption comparable to flour.

Despite its economic and cultural relevance, reliable massive genomic information (i.e. annotated transcriptomes / genomes) is not available for *Ilex paraguariensis*. In the absence of a sequenced genome, de novo assembly of RNA-Seq is a cost-effective method to study the transcriptome of most organisms [8]. RNA-Seq has enhanced our understanding of global gene expression and functions in plant kingdom, revealing novel sequences, transcript isoforms and single nucleotide polymorphisms (SNPs) [9]. In plants with large and complex genomes RNA-Seq has accelerated the discovery of novel genes, tissue-specific expression patterns and functional analysis. Debat et al. [10] reported a global expression assay in *I. paraguariensis* derived from a single RNA-Seq library. Nevertheless, a comprehensive and consistent transcriptomic data and experimentally validated expression profiling from different tissues are still lacking. In this study, we present a *de-novo* assembled and annotated transcriptome based on 15 libraries from different tissues of *Ilex paraguariensis*. Our findings provide a reasonably complete catalogue of the expressed genes in plant tissues along with functional annotation of those sequences, and experimental evidence of key *in-silico* predicted genes.

## Results

### RNA sequencing and transcriptome de novo assembly

To achieve a comprehensive representation and characterisation of the of *Ilex paraguariensis* transcriptome, we sequenced 15 RNA-seq libraries from four tissues, namely root, seedling, normal leaves and curly leaves (Additional file 1: Figure S1), thus maximising the number of active genes captured across tissues. The curly leaf phenotype is currently present in most of the YM trees associated with one of the main diseases of this crop [11].

**Fig. 1:**
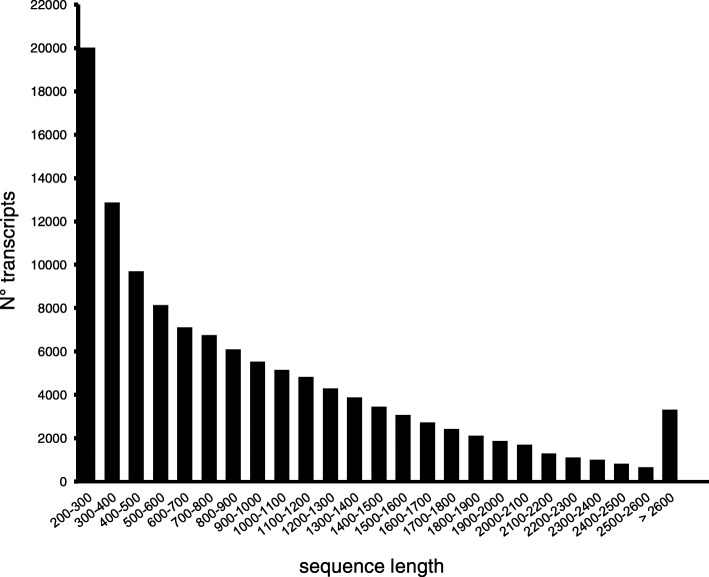
Length distribution of *Ilex paraguariensis* transcripts. X axis: sequence length in base-pairs, Y axis: number of transcripts featuring a particular length

The combined paired-end reads data sets from all the libraries − 554 million reads– contributed to build a reference de novo assembly, resulting in 470,638 contigs with mean contig length of 374 bp and N50 value of 766 bp (Table 1). Shorts contigs (< 200 bp) were filtered out remaining a total of 193,897 assembled transcripts ranging from 200 to 10,640 bp (Table 1), with a mean transcript length of 920 bp. Among these transcripts, 87,323 (45%) were longer than 500 bp. Transcripts length distribution is shown in Fig. 1.

**Table 1:** Summary of transcriptome data obtained from *Ilex paraguariensis leaves, root and seedlings*

Total N° of contigs	470,638
Mean contig length (bp)	374
Minimum contig length (bp)	101
Maximum contig length (bp)	10.640
Contig N50^†^ (bp)	766
Contig N80^†^ (bp)	1750
Contig N90^†^ (bp)	2260
Total bases	176,377,000
Standard Deviation of contig length (bp)	490.66
Unigenes > 200 bp	193,897
Unigenes > 500 bp	87,323
Mean unigen length	927 bp
Median unigen length	731 bp
Unigenes with orthologue in DBs	119,880
Unigenes in SwissProt Database	67,915
Unigenes in TrEMBL Database	51,965

† N50 value is defined as the contig length where half the assembly is represented by contigs of this size or longer; the N80 value is defined as the contig length where 80 % of the assembly is represented by contigs of this size or longer; N90 value is defined as the contig length where 90 % of the assembly is represented by contigs of this size or longer

The sequence reads quality analysis was performed with FastQC [12]. Averaged base quality per read was > 38, adapters and N sequence contents were negligible. We evaluated the completeness, quality and accuracy of the assembled transcriptome by (1) mapping the sequence reads back to the assembled contigs using TransRate [13], and (2) by assessing the presence of 1440 single-copy plant core genes using BUSCO v.3 [14]. After estimating the assembly score and read-mapping metrics as implemented in TransRate, we contrasted the outcome with 155 de novo transcriptome assemblies [13]. The resulting assembly score (0.17) placed our transcriptome assembly above 40% of the 155 assemblies, and outperformed the assembly scores of most (8 out of 10) of the representative assemblies evaluated by Smith-Unna et al. [13], including 3 rice assemblies. The proportion of read pairs that mapped back to the assembled transcripts (73%) is comparable to that reported for the 10 representative assemblies mentioned above. The TransRate read mapping count was also consistent with the *concordant_aligned* read pairs (73,26%) derived from RSEM analyses averaging all 15 RNA-Seq libraries. In terms of completeness, BUSCO results revealed that ~ 84% of the 1440 Embryophyte gene set are present in the current transcriptome assembly either as complete (73.5%) or fragmented genes (10.8%), with only a minor fraction of missed genes (15.8%). Hence, most of the orthologs were accurately identified as complete single copy (C:1058 [S:860, D:198], F:155, M:227, n:1440). Duplicated genes (13.7%) may result from multiple haplotypes, alleles or isoforms retained in the clustering analysis. Results from both, TransRate and BUSCO analyses are therefore indicative of a reasonably complete, consistent and good quality *Ilex paraguariensis* transcriptome assembly. The *Ilex paraguariensis* transcripts sequence data generated were deposited at NCBI as Transcriptome Shotgun Assembly (TSA) GEWR00000000, BioProject PRJNA315513, under accession numbers GEWR01000001 to GEWR01193693.

### Gene annotation and full-length transcript prediction

Sequence annotations of all transcripts were predicted using BLASTX against the UniProtKB database with an E value cut off of *10*^*− 6*^. A total of 119,880 unigenes (~62% of > 200 bp transcripts) showed significant similarity to known proteins, 67,915 (35.03%) in SwissProt and 51,965 (26.80%) in TrEMBL (Table 1); 61,218 unigenes (32%) remain unknown and require further investigations.

Transcripts with at least one BLAST hit (43%) showed > 70% sequence identity to a matching database sequence, indicating the reliability and quality of the assembled transcripts.

**Fig. 2:**
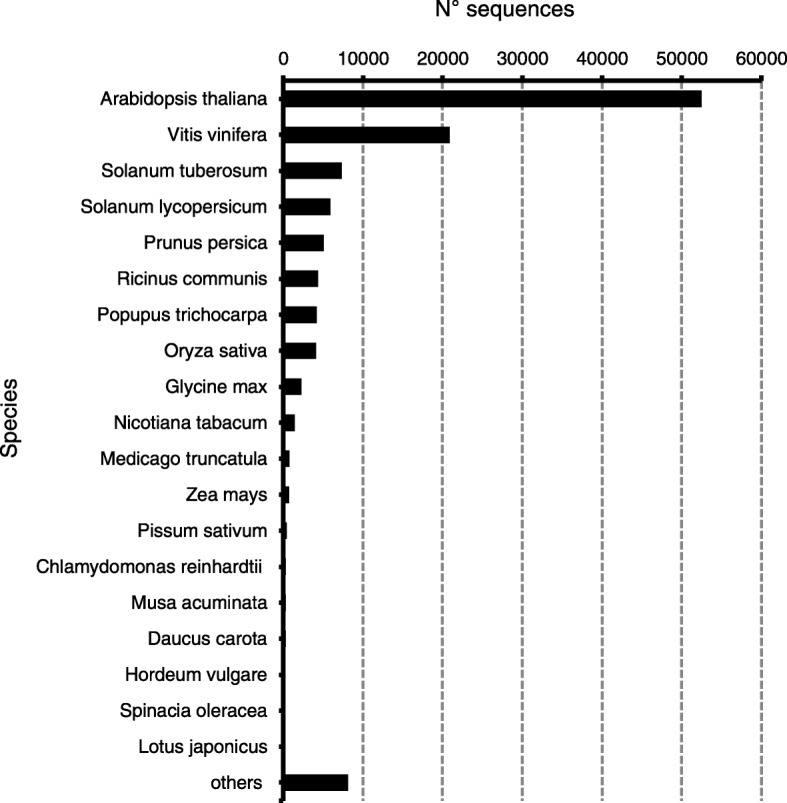
Transcriptome annotation: distribution of *Ilex paraguariensis* transcripts matching protein sequences of plant species available at UniProt database

In the species distribution analysis, 52,518 transcripts (43%) had the best match to *Arabidopsis thaliana* sequences, followed by 20,877 transcripts (17%) to *Vitis vinifera,* 7324 (6%) and 5891 (5%) to *Solanum tuberosum* and *Solanum lycopersicum* respectively (Fig. 2). Annotations are summarized in Additional file 2: Data S1A and Additional file 3: Data S1B.

Protein prediction accuracy increases with the ability to identify full-length cDNAs sequences. All transcripts were analysed by Full-lengther software to identify potential full-length cDNAs with complete open reading frame (ORF) in the assembled transcriptome of *Ilex paraguariensis*. In total, 24,612 (12.69%) unigenes were identified as full-length transcripts and 6863 (3.54%) as putative full-length (Table 2). Complete protein sequences corresponding to full-length transcripts derived from the transcriptome data are available in Additional file 2: Data S1A and Additional file 3: Data S1B.

**Table 2:** Full-lengther summary status of the *Ilex paraguariensis* transcriptome assembly

Status		Unigenes	%
Complete	Confirmed	24,612	12.69
	Putative	6863	3.54
C-terminus	Confirmed	30,440	15.70
	Putative	5076	2.62
N-terminus	Confirmed	11,598	5.98
	Putative	4338	2.24
Internal		36,358	18.75
Misassembled		595	0.31
Putative chimera		0	0.00
Coding	Confirmed	6184	3.19
	Putative	6522	3.36
Putative ncRNA		93	0.05
Unknown		61,218	31.57
*Total*		*193,897*	*100.00*

### Functional classification

**Fig. 3:**
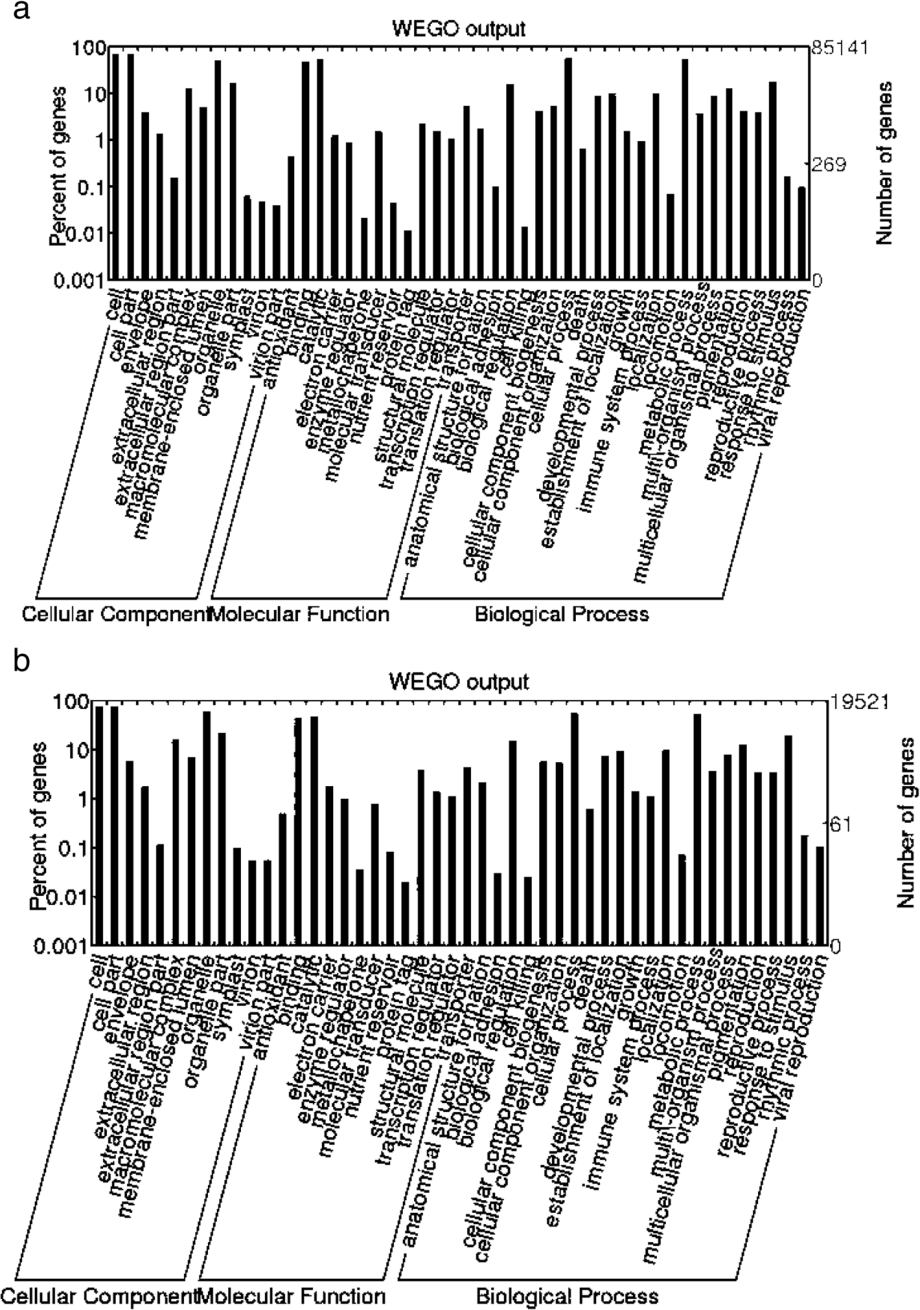
GO classification of unigenes derived from *Ilex paraguariensis*. Histograms show the classification of 85,141 unigenes (**a**) and 19,521 full-length annotated transcripts (**b**) into GO major categories (biological processes, cellular components, and molecular function) and 48 subgroups. Rigth Y-axis: number of unigenes. Left Y-axis indicates the percentage of specific categories of genes in each main category

Annotated transcripts were classified into three main independent GO categories: biological processes, molecular functions, and cellular components. A total 85,141 unigenes (71%) were associated with at least one GO term. Within molecular function category, genes involved in the binding and catalytic activities were highly represented. Under biological processes, main subcategories were cellular process, metabolic process and response to stimulus. Finally, most of the assignments cellular components were cell parts, cell and organelles (Fig. 3a).

In the functional classification of full-length transcripts, a total of 19,520 transcripts (79%) presented at least one GO term, showing a distribution of unigenes in each category similar to that observed in the analysis including the full set of transcripts (Fig. 3b).

### Kyoto Encyclopaedia of genes and genomes (KEGG) classification

**Fig. 4:**
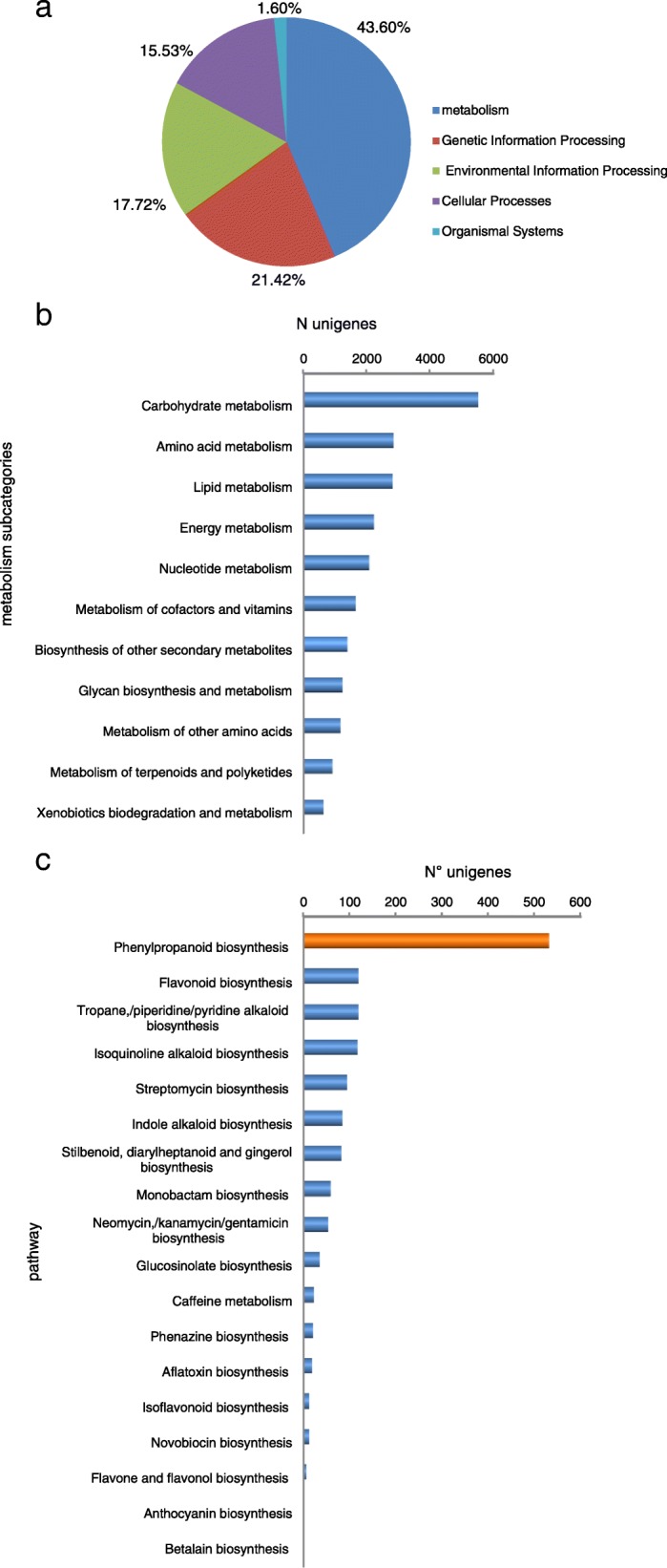
Distribution of the unigenes derived from *Ilex paraguariensis* in KEGG pathways: unigenes were classified into five main categories (**a**); into subcategories within Metabolism (**b**); and in Secondary Metabolites Enzymatic Pathways within Metabolism (**c**). Note the relevance of the Phenylpropanoid pathway related genes identified in this work (orange bar)

**Fig. 5:**
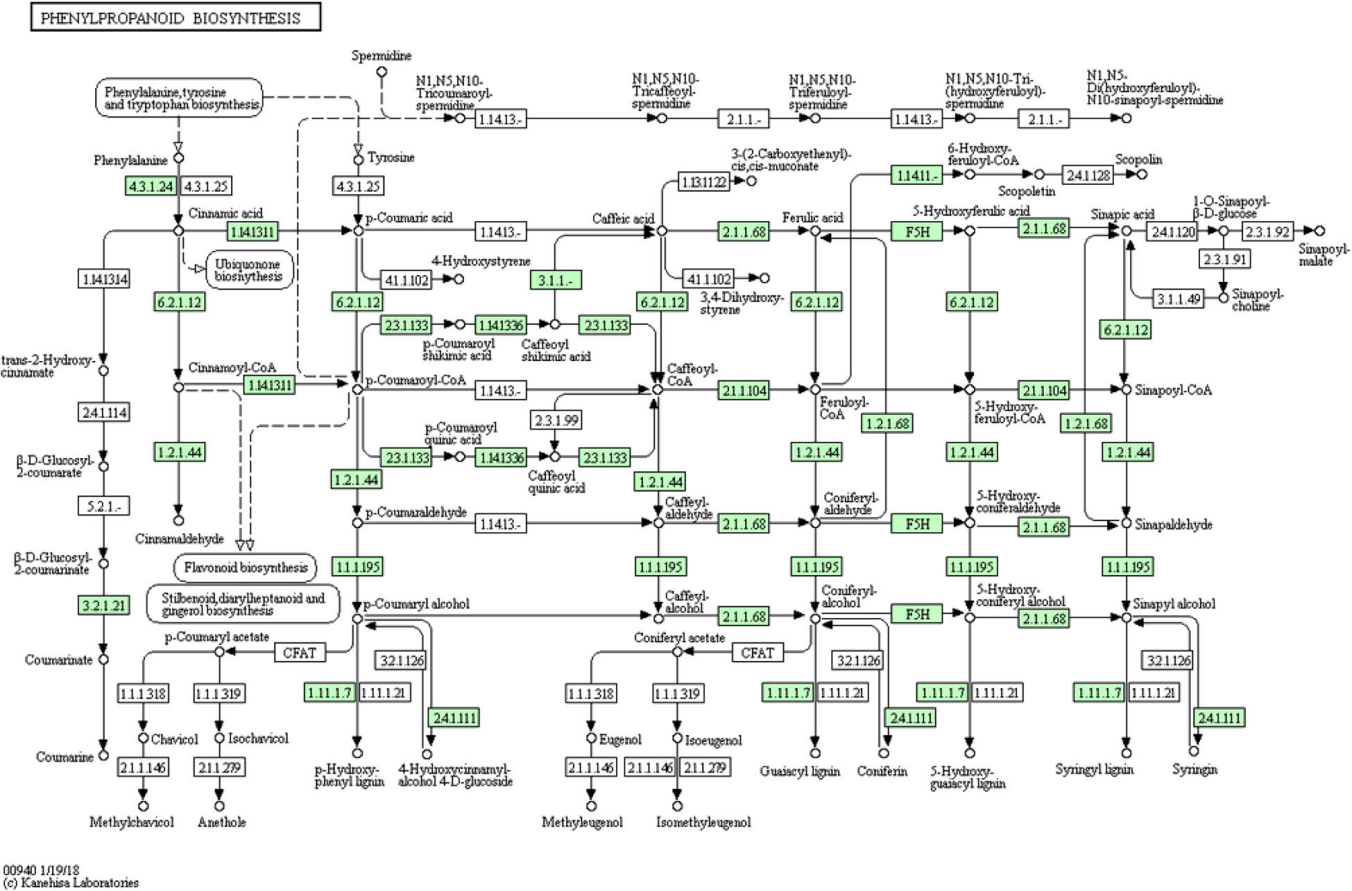
The Phenylpropanoid pathway: *Ilex paraguariensis* transcripts encoding enzymes involved in this pathway identified by KEGG annotation are highlighted in green. The total number of unigenes coding for each enzyme is summarised in Table 3

Pathway based analysis help us to further understand the transcription activity and biology behind specific compounds of interest. Unigenes were blasted against the KEGG pathway database. In total, 58,987 (44%) out of 119,880 unigenes were identified and assigned into 228 KEGG pathways covering five major categories including Metabolism, Cellular Processes, Genetic Information Processing, Environmental Information Processing and Organismal Systems. Metabolism (22,503 unigenes, 38%) was the most represented category, with metabolism of carbohydrate, amino acids and lipids as the outstanding pathways within it (Fig. 4a). Interestingly 1395 unigenes are involved in the biosynthesis of secondary metabolites (Additional file 4: Table S1) and many of them, such as phenolic compounds, chlorogenic acids and flavonoids, are responsible for the *Ilex paraguariensis* health related benefits. Polyphenols, synthesized through the Phenylpropanoid pathway, have shown a strong correlation to its overall antioxidant capacity [15]. KEGG annotation assigned 531 transcripts to the phenylpropanoid pathway, being the most frequently represented biosynthetic pathway among secondary metabolites. From these transcripts, 142 corresponded to full-length transcripts (Table 3). The three initial steps of the phenylpropanoid pathway catalyzed by PAL, cinnamate 4-hydroxylase, and 4-coumaroyl CoA-Ligase are mandatory and provide the basis for all subsequent branches and resulting metabolites. We identified these transcripts including phenylalanine ammonia lyase (PAL, EC 6.2.1.12, 28 unigenes), cinnamic acid 4-hydroxylase (C4H, EC 1.14.13.11, 6 unigenes), and 4- coumarate-CoA ligase (4CL, EC 6.2.1.12, 21 unigenes), experimentally validating PAL and 4CL. In addition, core enzymatic components of the flavonoid and flavonol biosynthesis pathways were observed in the transcriptome we generated, including Chalcone synthase (CHS,EC 2.3.1.74 unigene), Chalcone isomerase (CHI,EC 5.5.1.6, unigene), flavonoid 3'- monoxygenase (F3’H, EC 1.14.13.21 unigene), flavonol synthase (FLS, EC 1.14.11.23 unigene) (Table 4, Fig. 5).

**Table 3:** *Ilex paraguariensis* transcripts assigned to secondary metabolism biosynthesis according to KEGG annotation: *Phenylpropanoid biosynthesis*

KO id	Description	N° transcripts	N° full-length transcripts
K10775	phenylalanine ammonia-lyase	28	4
K01188	beta-glucosidase	200	49
K01904	4-coumarate--CoA ligase	21	6
K09753	cinnamoyl-CoA 4-monooxygenase	8	2
K00487	trans-cinnamate 4-monooxygenase	6	2
K12355	coniferyl-aldehyde dehydrogenase	8	2
K00083	cinnamyl-alcohol dehydrogenase	30	14
K11188	peroxiredoxin	9	0
K00430	peroxidase	99	29
K12356	coniferyl-alcohol glucosyltransferase	8	2
K13065	shikimate O-hydroxycinnamoyltransferase	36	19
K09754	coumaroylquinate 3′-monooxygenase	12	2
K18368	caffeoylshikimate esterase	5	2
K13066	caffeic acid	31	5
K00588	caffeoyl-CoA O-methyltransferase	23	4
K09755	ferulate-5-hydroxylase	3	0
K06892	feruloyl-CoA ortho-hydroxylase	4	0
TOTAL		531	142

Transcripts identified in the Phenylpropanoid pathway (A) and in the Flavonoids pathway (B)

**Table 4:** *Ilex paraguariensis* transcripts assigned to secondary metabolism biosynthesis according to KEGG annotation: *Flavonoid biosynthesis*

KO id	Description	N° transcripts	N° full-length transcripts
K00660	chalcone synthase	7	2
K01859	chalcone isomerase	5	2
K00475	naringenin 3-dioxygenase	2	2
K05278	flavonol synthase	13	2
K00487	trans-cinnamate 4-monooxygenase	6	2
K05280	flavonoid 3′-monooxygenase	2	0
K05277	leucoanthocyanidin dioxygenase	4	0
K08695	anthocyanidin reductase	1	0
K13080	flavanone 7-O-glucoside 2”-O-beta-L-rhamnosyltransferase	3	3
K13065	shikimate O-hydroxycinnamoyltransferase	36	19
K09754	coumaroylquinate(coumaroylshikimate) 3′-monooxygenase	12	2
K13082	bifunctional dihydroflavonol 4-reductase/flavanone	6	0
K00588	caffeoyl-CoA O-methyltransferase	23	4
*Flavone and flavonol biosynthesis*			
K13264	isoflavone 7-O-glucoside-6”-O-malonyltransferase	1	1
K13080	flavanone 7-O-glucoside 2”-O-beta-L-rhamnosyltransferase	3	3
K05280	flavonoid 3′-monooxygenase	2	2
K13272	flavonoid O-methyltransferase	1	0
*Anthocyanin biosynthesis*			
K12930	anthocyanidin 3-O-glucosyltransferase	2	2
*Isoflavonoid biosynthesis*			
K13258	2-hydroxyisoflavanone dehydratase	2	0
K13260	isoflavone 2′-hydroxylase	5	0
K13263	isoflavone 7-O-glucosyltransferase	2	1
K13265	vestitone reductase	4	0
TOTAL		142	47

Transcripts identified in the Phenylpropanoid pathway (A) and in the Flavonoids pathway (B)

### Experimental validation of assembled transcript

To experimentally validate the reliability of the unigenes obtained from the assembled transcriptome, we PCR-assayed 11 unigenes potentially involved in photosynthesis, stress response, polyphenol synthesis and general cell cycle activity. Selected PCR primers generated single specific amplicons of the expected size (Additional file 5: Figure S2). Sequencing results confirmed transcription in leaves of genes involved in photosynthesis (i.e. plastocyanin (*PC*), oxygen evolving enhancer (*OEE*)), in stress response (i.e. lipid transfer (*LTP*), peroxidase (*PX*), major allergen (*MAL*), metallothionein (*MET*)), and in general cell cycle activity (i.e. ubiquitin (*UBQ*), alpha tubulin (*TUA*); as well as three unigenes associated with the phenylpropanoid pathway: phenylalanine ammonia-lyase *(PAL),* chalcone synthase (*CHS*) and 4 coumarate-ligase (*4CL*).

### Expression profiles of transcripts in different *Ilex paraguariensis* tissues

**Fig. 6:**
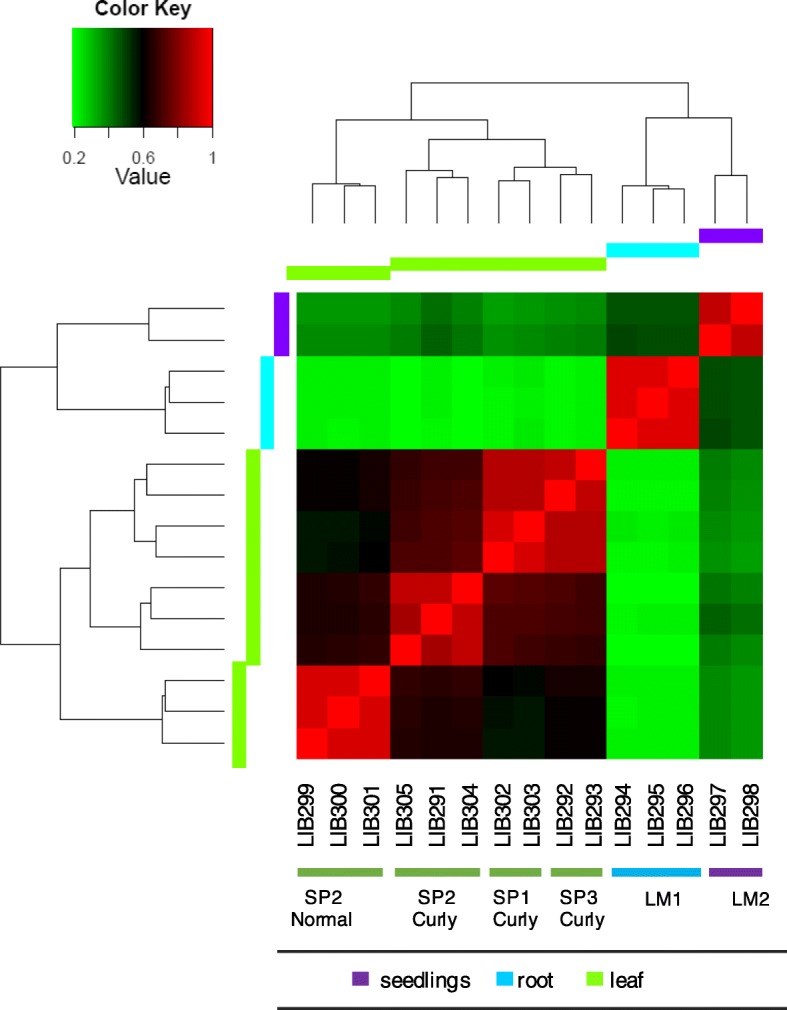
Correlation plot of the global gene expression profiles in different tissues of *Ilex paraguariensis*. The heatmap indicates consistency in the expression profile clustering from normal leaves (tree SP2) and curly leaves (trees SP2, SP1, SP3) (green), root (blue), and seedlings (purple) RNA-Seq libraries. Correlation was calculated using Euclidean’s distance matrix using DESeq2 package and the colour scale indicates the degree of correlation between sequenced libraries. Refer to Table 5 to look up the corresponding sample ID for each library

**Fig. 7:**
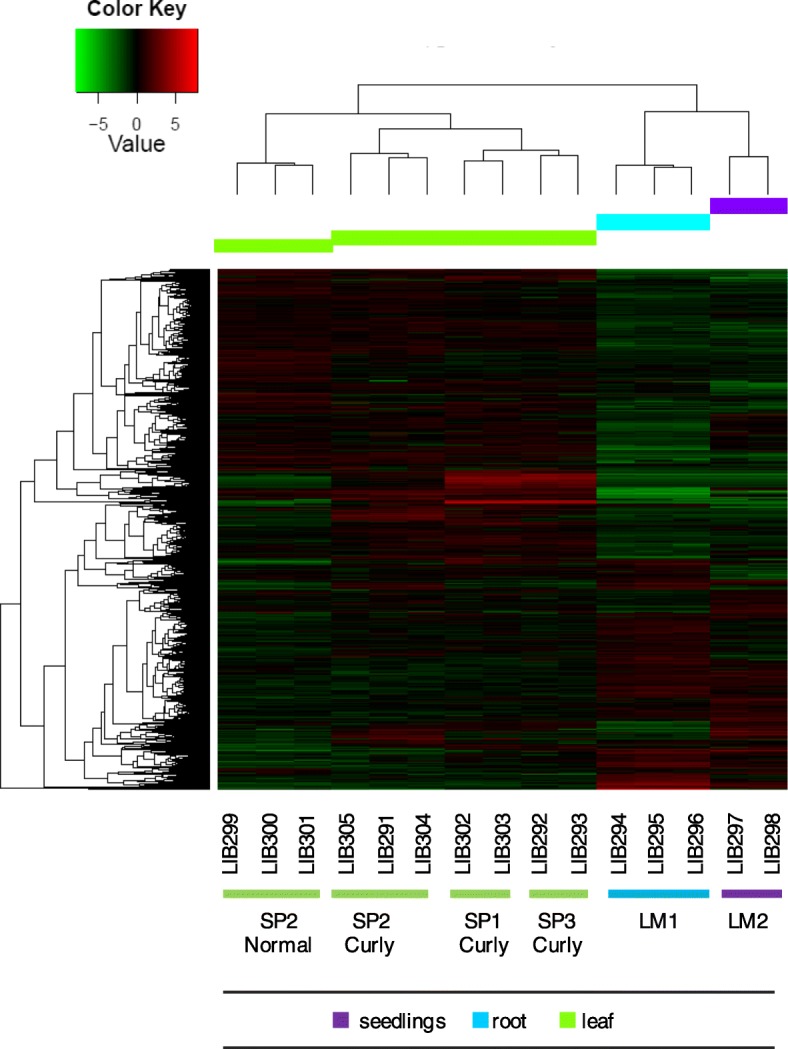
Differential transcriptional activity in *Ilex paraguariensis* tissues revealed by global transcription profiles. Each line corresponds to a single transcript in 15 RNA-Seq libraries (columns) derived from different tissues. Red and green lines indicate up-regulated and down-regulated transcripts respectively. The scale on the top left depicts the level of transcript expression. The dendrogram on the top denotes consistency in the clustering of libraries derived from normal and curly leaves (green), root (blue), and seedlings (purple). Refer to Table 5 for the corresponding sample ID for each library

Global expression profiling provides a key insight into the different on-going cellular processes under various conditions. To estimate expression abundance for transcripts across tissues of *Ilex paraguariensis*, paired-end reads from each sample were aligned to the de novo transcriptome assembly, measuring transcript expression as direct count and the FPKM (fragments per kilobase of exon per million mapped fragments) values. Approximately 81% of reads from each library could be mapped back to the originally assembled transcripts (Additional file 6: Table S2). From the normalized reads counts (log transformation) we generated a Euclidean’s distance matrix to compare the expression patterns among the 15 RNA-Seq libraries and to statistically evaluate the relationships among analysed samples and correlations among experimental replicates. The correlation plot in Fig. 6 illustrates the global relationship among all RNA-Seq libraries showing the clustering of libraries according to the tissue type, and that experimental replicates are indeed highly correlated. The clustering analysis in Figs. 6 and 7 clearly highlight (a) the presence of differentially expressed genes among tissue type, and (b) the presence of smaller but consistent differences between normal vs curly leaves collected from a single individual (SP2, Table 5). The dendrogram showed two major clusters according to the origin of the tissue, one that includes all leaves’ libraries and another one that comprises two different sets discriminating root samples from seedlings (whole plant). Therefore, each of these groups contains tissue-specific libraries.

**Table 5:** Sampled individuals and corresponding RNA Seq libraries

Collection site	Individual	Sample ID	Tissue	Replica	RNA Seq Library ID
SANTO PIPO	SP1	N°739	LEAF *Curly*	*a*	LIB8302
			LEAF *Curly*	*c*	LIB8303
	SP2	N°738	LEAF *Normal*	*a*	LIB8299
			LEAF *Normal*	*b*	LIB8300
			LEAF *Normal*	*c*	LIB8301
		N°740	LEAF *Curly*	*a*	LIB8304
			LEAF *Curly*	*b*	LIB8305
			LEAF *Curly*	*c*	LIB8291
	SP3	N°741	LEAF *Curly*	*a*	LIB8292
			LEAF *Curly*	*b*	LIB8293
LAS MARIAS	LM1	N°737	ROOT	*a*	LIB8294
			ROOT	*b*	LIB8295
			ROOT	*c*	LIB8296
	LM2	N°742	WHOLE Plant	*a*	LIB8297
			WHOLE Plant	*b*	LIB8298

Significant differentially expressed genes among *Ilex paraguariensis* tissues were identified. Only those unigenes showing four-fold change in expression (*p adjusted*-value < 0.001) were considered differentially expressed including 19,909 transcripts in normal leaf as compared to root, where 11,332 and 6795 genes were up- and down-regulated in leaf, respectively. When comparing global transcription activity in curly leaves vs normal leaves from the same tree (Table 5, individual SP2, samples N°738 vs 740) we found 13,249 transcripts showing significant differential expression. This observation is supported by six RNA-Seq libraries corresponding to three experimental replicas made for each of the two samples (curly and normal leaves) collected from a single individual (Fig. 7, Table 5).

**Fig. 8:**
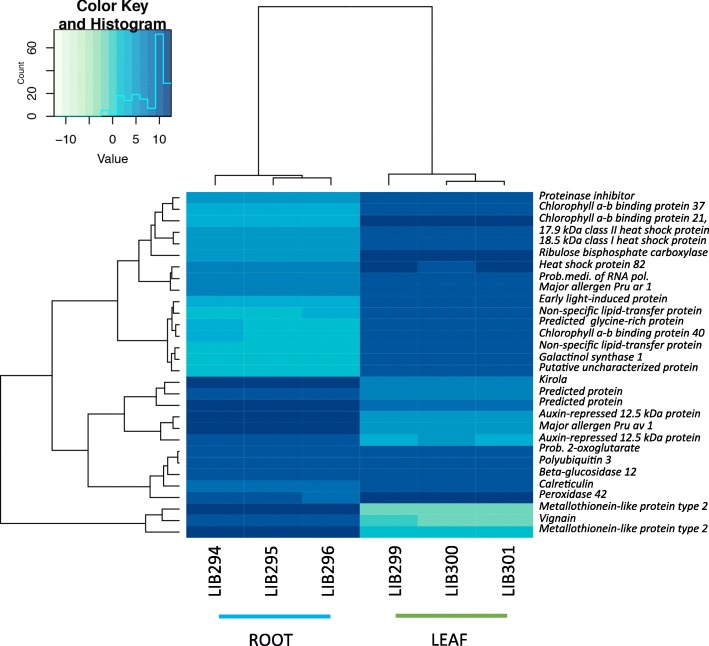
Contrasting expression in leaf vs root of *Ilex paraguariensis.* The heatmap shows 30 transcripts presenting the most divergent expression in RNA-Seq libraries from leaves and roots (columns). The transcript annotation (right column) can be used to retrieve the protein data and sequence from the protein annotation table: Additional files 2 and 3: Data S1A and S1B

KEGG pathway-based analysis of transcripts showing significant differential expression between leaf and root tissue revealed enriched metabolic pathways resulting from up regulated transcripts. The heatmap in Fig. 8 shows the 30 top transcripts with the most contrasting differential expression –leaf vs root– where the main block of up-regulated transcripts in leaf contains encoding proteins genes involved in photosynthesis such as chlorophyll binding protein, oxygen evolving enhancer, light-harvesting complex, and heat shock proteins. There are two smaller blocks of up-regulated genes in root related to auxins, methallothionein and major allergen that are involved in stress response.

### Quantitative real-time PCR (qRT-PCR) analysis

**Fig. 9:**
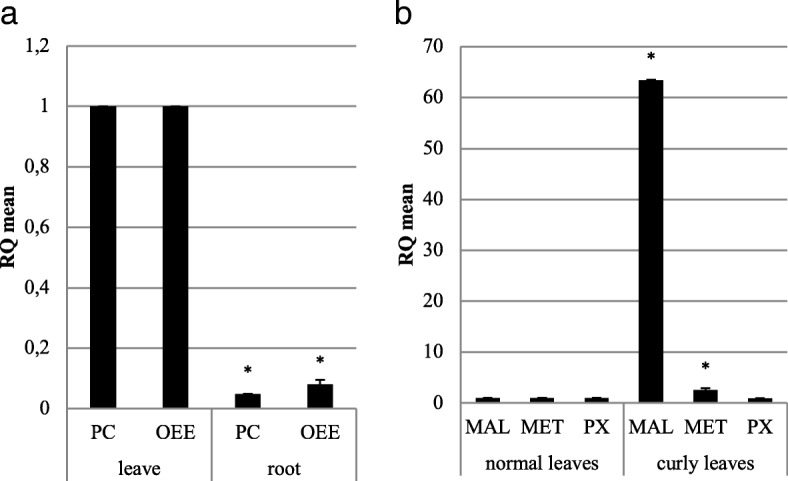
Relative gene expression (2∧-ΔΔCT) estimates using real time RT-PCR in five genes showing differential expression profiles in DESeq analyses of in *I. paraguariensis* RNA-Seq libraries. **a** Plastocianin (*PC*) and oxygen evolving enhancer (*OEE*) genes confirmed higher expression in leaves compared to root. **b** Major allergen (*MAL*) and metallothionein (*MT*) are highly expressed in curly leaves but not in normal leaves. Peroxidase (*PX*) transcriptional activity did not significantly differed between leaves samples. Y-axis depicts relative expression levels as 2-ddCt (RQ). X-axis details genes, different tissues and samples. Mean plotted values were obtained from 2 independent experiments. * indicates significantly different dCT values (RQ < 0.5 or RQ > 2)

We conducted qRT-PCR experiments to validate differential gene expression profiles revealed by DESeq analyses. From the ten genes used to evaluate the assembly (Additional file 5: Figure S2), we selected five that presented significant differential expression between tissue types in the DESeq analyses. Specifically, we investigated the expression levels of two genes involved in photosynthesis coding for plastocyanin (*PC*) and oxygen evolving enhancer (*OEE*), in three genes associated with response to stress, *i.e*, major allergen (*MAL*), metallothionein (*MET*) and peroxidase (*PX*), using tubulin A (*TUA)* as reference gene. As shown in Fig. 9a, transcriptional activity assayed in two photosynthesis related genes in normal leaf and root is consistent with the global expression data obtained by RNA-Seq, for instance, the expression of *PC* and *OEE* was higher in leaves. On the other hand, when comparing curly vs normal leaves (Fig. 9b), two stress response genes showed, as expected, significant contrasting expression levels (*MAL* and *MET*) being up-regulated in curly leaves, while differences in peroxidase (*PX*) expression were not significant.

## Discussion

Yerba Mate (*Ilex paraguariensis A. St. Hil.*) is the most relevant regional socioeconomic and cultural product in northeast Argentina, gathered and cultivated for centuries by aboriginal people and lately extended by European settlers [16]. The increasingly interesting properties of this widely spread infusion as antioxidant, anti-inflammatory, antimicrobial [3, 17, 18], make Yerba Mate a valuable resource for human health. Genomics tools, however, have not yet been effectively incorporated in Yerba Mate biotechnological applications likewise other crops. In this study, we have successfully assessed transcription activity in different tissues of *Ilex paraguariensis* –i.e. leaves, root and seedlings– releasing the sequence of > 24,000 full length transcripts not previously described in Yerba Mate and experimentally validating annotated (predicted) genes. We substantially increased the wealth and consistency of the genomic data in *Ilex paraguariensis,* setting a new reference for global expression analyses. Our work contributes with multiple RNA-Seq libraries derived from main tissues with experimental replicas, providing a more comprehensive coverage of gene expression activity and full-length transcripts. Our entire transcript list is readily accessible in public domain without the need to re-assemble and re-annotate the raw sequence read data. Additionally, we have also evaluated transcripts assembly consistency by PCR amplification and sequencing exonic regions of 11 predicted genes.

The average length for 193,897 transcripts assembled from > 500 million high-quality reads was 920 bp, comparable to transcriptomic analyses in other plant species [19–22]. A large number of transcripts (62%) were successfully aligned to known proteins in Swiss-Prot database and the GO classifications into main GO annotation subcategories were similar to those of other species as well [21, 23]. Main functions of annotated transcripts are therefore assigned to metabolism common in plants.

Tissue specific gene activity has been verified in several plant species, here we report global expression profiles in *Ilex paraguariensis* that can clearly identify significant differentially expressed transcripts among tissues. Both, the correlation plot among RNA-Seq libraries (Fig. 6) and the clustering of transcriptional activity (Fig. 7) show consistent differences among tissue global expression profiles. This contrasting gene activity in root and normal leaves has been experimentally verified by qRT-PCR in selected genes (Fig. 9a). These results highlight the contribution of each tissue to the general transcriptome assembly.

By assessing differential gene expression among tissues we aimed both, (a) to validate this hypothesis as a proof of principle for further experiments, and (b) to set the context to investigate potential differences between the two leaf phenotypes. Disparities in gene expression levels between normal and curly leaves are evident in Fig. 7. Seven RNA-Seq libraries (Fig. 7, columns 4–10) derived from 3 curly leaves samples (Table 5, samples N° 739, 740, 741) presented a global expression profile that can be clearly distinguished from three RNA-Seq libraries (Fig. 7, columns 1–3) derived from a normal leaf sample (Table 5, sample N°738). Divergent gene activity in these particular genes may result from either, distinctive genetic backgrounds or from plant response to dissimilar environmental conditions. However, all leaves samples were collected the same day from a single collection site (Santo Pipo, < 2,5Ac). Furthermore, the expression profiles in LIB299, LIB300 and LIB301 (Fig. 7 columns 1–3) and those profiles in LIB305, LIB291, LIB304 (Fig. 7, columns 4–6) actually represent two samples of the same individual (SP2) differing only on the leaf phenotype (Table 5). Using qPCR we looked at the transcriptional activity of three genes involved in stress response that had high expression in the RNA-Seq profiling and corroborated significant differential gene expression between normal and curly leaves in two of them (Fig. 9b). Four additional RNA-Seq libraries from curly leaves collected from another two individuals (SP1, and SP3) at the same collection site resulted in expression profiles similar to the first curly leaf evaluated (SP2). Curly leaf is a particular phenotype associated with a widespread disease in *Ilex paraguariensis* that severely impairs yerba mate production [11]. We hypothesised that differences in the expression profiles of normal vs curly leaves could potentially be related to the disease. Further studies are necessary to investigate the presence of potential pathogens in curly leaves and to experimentally assess gene expression comparing normal vs curly leaves of single plants in large numbers during the disease season. We identified the top 30 differentially expressed transcripts in RNA-Seq libraries from different tissues, and experimentally validated few of them recognising genes involved in plant immunity and synthesis of antioxidant compounds. MAPK signalling pathway and plant-pathogen interaction were well represented in the pathway analysis from up-regulated transcripts in leaves from different individuals. Transcriptional activity in this pathway is triggered by different biotic and abiotic stress stimuli such as pathogen infection [24].

Transcriptome analysis has been extensively used to unravel genes encoding enzymes involved in key biosynthetic pathways steps of active compounds in medicinal plants [25]. We validated and further investigated the activity of genes responsible for antioxidant mechanisms involved in the phenylpropanoid pathway. *Ilex paraguariensis* is a potential source of phenolic compounds with widely known biological properties. Considering the top 30 plant species of industrial interest with the highest records of antioxidant activity, dry leaves aqueous extract sets *Ilex paraguariensis* in the fifth position [26]. These remarkable antioxidant properties are attributed to its high polyphenol concentration synthesized through the phenylpropanoid pathway [27], and contribute to key functional aspects of plant life such as UV sunscreens, pigments signalling and plant immunity [28]. Interestingly, accumulation of phenolic compounds is stimulated by biotic and abiotic responses [29]. We identified 531 KEGG annotated transcripts potentially involved in the phenylpropanoid pathway of *Ilex paraguariensis* (Additional file 4: Table S1), and experimentally validated the expression of key genes not reported before in Yerba Mate and known to have a relevant role in other plants. Transcript sequences sharing identical KEGG Orthology terms (KO, Additional file 4: Table S1), may result from either isoforms, alternate splicing and multi-copy gene family; *i.e* phenylalanine ammonia-lyase gene family codes for > 25 genes in tomato (*Lycopersicon esculentum*) and 4 genes in Arabidopsis (*Arabidopsis thaliana*) –*PAL1-PAL4–* with different gene structure [30–32]. The number of PAL related transcripts observed confirms the presence and activity of PAL gene family members. Furthermore, the alignment of PAL related transcripts (cDNA) and DNA sequences derived from a 1200 bp PCR amplicon indicates the existence of different genes sharing a fragment of at least 350 bp. Additional work is necessary to precisely identify the actual number and activity of PAL genes in *Ilex paraguariensis*.

## Conclusions

Our results provide a reliable and comprehensive transcription profile of *Ilex paraguariensis,* and details of previously unknown genes in this species with experimentally validated sequence and activity, which are relevant for production traits, including compounds content. Having annotated transcripts covering most of the components of the Phenylpropanoid pathway, it is now possible to focus on these candidate genes to study variation in the amount of polyphenol in leaves. These data offer an essential resource to further explore genes involved in metabolism of antioxidant compounds and disease tolerance, and drive future studies targeting genes underlying relevant agronomic traits for this emerging crop.

## Methods

### Tissue samples and RNA isolation

Tissue samples of *Ilex paraguariensis* such as normal and curly leaves from adult trees, roots and seedlings (whole-plant) were collected from two collection sites in Argentina: Santo Pipó, Misiones, and Las Marías, Corrientes. All leaves samples belong to 3 individuals (SP1, SP2, SP3, Table 5) of similar age raised in the same parcel at a single collection site (Santo Pipó); collected in a single day within two hours and immediately frozen in liquid nitrogen until proceeding with RNA isolation up to two days after collection. From individual SP2, two leaves samples were collected, one with normal leaves (sample N°738) and the other sample containing curly leaves (sample N°740). Curly leaves are present in the vast majority of YM trees at almost any parcel as a symptom of a widespread disease produced by *Gyropsylla spegazziniana* (Psyllidae) (Additional file 1: Figure S1). Total RNA was isolated from each sample using Purelink RNA Mini Kit (Ambion) and in-column treatment with RQ1 RNase-Free DNase (Promega) to remove any source of DNA contamination. RNA integrity and purity were evaluated in 1% agarose gel electrophoresis and using a Nano-MaestroGen spectrophotometer (GE). Before the cDNA library preparation, RNA quality was assessed using 2100 Agilent Bioanalyzer to determine RNA integrity number (RIN). We then set three experimental replicas from each sample and a single RNA-Seq library was generated for each replica (Table 5). Only curly leaves were collected from individuals SP1 and SP3 with two experimental replicas / RNA-Seq library each. The root sample (LM1) and the whole plant sample (LM2) were obtained from two individuals in a seedling tray before they were transplanted to the main field in Las Marías.

### cDNA library preparation and transcriptome sequencing

The high quality RNA extracted from each tissue sample (RIN > 6, RNA concentration 20 ng/ul, total RNA) was employed for the generation of 15 cDNA libraries using TruSeq RNA Sample Preparation Kit (Illumina Inc.). Each RNA-Seq library corresponds to an experimental replica as detailed in Table 5. cDNA libraries were then sequenced in two lanes of the Illumina HiSeq 2500 sequencing platform by paired-end sequencing (100 bp). High throughput sequencing was performed at The Genome Analysis Centre (TGAC) Norwich, UK (currently The Earlham Institute).

### Sequence assembly and annotation

Sequencing reads were pre-processed to remove adapters and low quality sequences. Qualified reads from all libraries were then assembled into contigs using the Trinity assembler method with default parameters [8]. Short contigs (< 200 bp) were filtered out and the remaining contigs were then connected into transcripts sequences. We used CD-HIT-EST to cluster transcripts at 90% identity. CD-HIT software is employed to decrease transcript redundancy in de novo transcriptome assemblies from different individuals, collections of plant tissues and in cases of high heterozygosity and polyploidy [33–37]. To evaluate assembly quality and accuracy we estimated the proportion of read pairs mapping to the assembled transcripts and the associated assembly score using TransRate [13]. Transcriptome completeness was assessed using the Benchmarking Universal Single-Copy Orthologs (BUSCO) tool [14]. BUSCO interrogates the assembled transcriptome gene content searching for a set of conserved single-copy orthologs derived from OrthoDB, and reports the proportion of complete, duplicated, fragmented, and missing genes in the assembly. We evaluated the transcriptome completeness performing the BUSCO v.3 [38] assessment that incorporates 1440 single-copy orthologous genes as the embryophyte dataset.

The transcripts assembly was compared with a set of full-length plant protein sequences database (UniProtKB, www.uniprot.org) to extend transcripts using Full-length program [39] and to assign putative functions to assembled transcripts (E-value <1E–05). The Gene Ontology (GO) terms were obtained from BLASTX against the Swiss-Prot database using Blas2GO program [40] to classify function assigned to the transcripts. The distribution of unigenes into functional categories was summarized using WEGO software [41]. Orthologous assignment and mapping of the unigenes to the biological pathways were performed using KEGG automatic annotation server (KAAS) with threshold bit-score default value of 60 (http://www.genome.jp/kegg/).

### Experimental validation of assembled transcripts

To check the reliability of transcripts reconstructed in silico, we designed primers using transcripts as templates to generate single specific amplicons of known size, and then PCR-amplified genes potentially involved in photosynthesis, stress response and general cell cycle. Specific primers were designed to amplify fragments of 100 bp from genes involved in photosynthesis (i.e. plastocyanin (*PC*) and oxygen evolving enhancer (*OEE*)), in stress response (i.e. lipid transfer (*LTP*), peroxidase (*PX*) and major allergen (*MAL*), metallothionein (*MET*)), polyphenols synthesis (Phenylalanine ammonia-lyase *(PAL),* Chalcone synthase (*CHS*) and 4 coumarate-ligase (*4CL*)), and general cell cycle activity (i.e. ubiquitin (*UBQ*) and alpha tubulin (*TUA*)), using the Primer Express software v.3.0. Total RNA was extracted from leaves, and cDNA was synthesised by reverse transcription. PCR fragments were purified and sequenced in both directions using the Sanger method. Consensus sequences from each amplicon were then “blasted” (BLASTn) against public data in GenBank. PCR / sequencing primers are listed in Additional file 5: Figure S2.

### Differential gene expression analysis

RNA-Seq data from sequenced libraries were used to assess differences in gene expression among samples. To estimate the expression pattern of each transcript in different tissues, reads from each cDNA library were mapped onto the final transcriptome assembly using Bowtie 2 package [42]. The read counts were then estimated for each transcript at individual libraries using RSEM software [43]. Differential gene expression analysis was performed using DESeq v1.14.1 software [44] by pairwise comparisons between root samples, seedlings, normal leaves and curly leaves. A *P*-value cut-off of ≤ 0.001 along with at least four-fold-changes were used to identify significant differential expression. To further evaluate the clustering of individual samples and experimental replicates, principal component analysis was performed by DESeq2 program using transformed count read data (log transformation).

### Quantitative real-time PCR (qRT-PCR) analysis

To verify the expression patterns revealed by DGE results, we performed quantitative real-time reverse transcription PCR (qRT-PCR) in transcripts showing significant differential expression patterns between tissue samples in the DESeq analyses, specifically *PC, OEE, MAL, MET, PE,* and *TUA* as a reference gene. Primers are listed in Additional file 5: Figure S2. Total RNA was extracted from a seedling (root, leaf) and from an adult tree (normal leaf, curly leaf) using Purelink RNA Mini Kit (Ambion) as described above. One microgram RNA was used to generate cDNA with the reverse transcription system kit (Promega) according to manufacturer’s guidelines. Real-time PCR was performed using Power SYBR Green PCR Master Mix and the StepOne detection system (ThermoFisher). The total mixture reaction volume (12 μL) contained 1.2 μL cDNA, Template (< 100 ng), 6 μL SYBR Premix (2X), 0.6 μL of forward and reverse primers (10 pmol) and 4.5 μL of dH2O (sterile distilled water). PCR conditions were as follows: 95 °C for 30s initial denaturation, followed by 40 cycles of denaturation at 95 °C for 5 s and annealing at 60 °C for 30s. All reactions were carried out in duplicated for technical and biological repetitions, and the amplicons were subject to melting curve analysis to determine amplification specificity. Raw data on the relative abundance of each transcript were expressed as mean ± standard deviation (SD). The relative expression levels of selected genes were normalized to the tubulin A gene using the 2-ΔΔCt method and data analysed using the Step One Software v2.2.2. Expression stability from internal control gene was assessed by the cycle threshold (Ct) values obtained in real-time PCR in a set of three different tissue samples (root, normal leaf and curly leaf). Prior to the 2-ΔΔCt analysis, we checked the efficiencies of the reference gene (*TUA*) and target genes (*PC, MET, PX, OEE, MAL*) were similar [45].

## Additional files

Additional file 1: **Figure S1.** Tissue samples and starting material. **A**: Samples were collected from different tissues to maximise the transcriptional activity coverage: normal leaves, curly leaves, seedling and root. **B**: Extent of the damaged caused by curly leaves on the tip of a single branch (*Ilex paraguariensis*) (PDF 2741 kb)

Additional file 2: **Data S1A.** Transcriptome annotation, protein sequences and prediction of full-length transcripts with complete ORF. Lines contain the amino acid sequence and relevant protein information corresponding to 119,880 transcripts > 200 bp annotated against reference databases (UniprotKB, SwissProt and TrEMBL). Note that among the 119,880 annotated transcripts we report 24,612 protein sequences with complete ORF derived from full length cDNA sequences. (XLSX 11123 kb)

Additional file 3: **Data S1B.** Transcriptome annotation, protein sequences and prediction of full-length transcripts with complete ORF. Lines contain the amino acid sequence and relevant protein information corresponding to 119,880 transcripts > 200 bp annotated against reference databases (UniprotKB, SwissProt and TrEMBL). Note that among the 119,880 annotated transcripts we report 24,612 protein sequences with complete ORF derived from full length cDNA sequences. (XLSX 10432 kb)

Additional file 4: **Table S1.** List of 531 annotated transcripts matching genes involved in the phenylpropanoid pathway according to the KEGG mapping. Each KEGG mapped transcript was associated to a KEGG Orthology term (KO). The column on the right shows the enzyme entry code (EC). Note that a number of transcripts share identical KO terms and EC indicating the presence of isoforms, splicing and multi-copy gene family as occurs in phenylalanine ammonia-lyase genes. (TXT 78 kb)

Additional file 5: **Figure S2.** Experimental validation of *Ilex paraguariensis* transcriptome assembly. PCR primers, PCR products and sequence identity of amplified cDNA sequences of 11 predicted genes in the *Ilex paraguariensis* transcriptome. **A**: List of 11 genes identified in the transcriptome annotation, the corresponding PCR primers sequence and expected amplicon size. **B**: PCR products matching expected size: 100, 150 and 1200 bp. **C**: Amplicon DNA sequence BLASTx results showing significant sequence identity to DNA sequences from other species deposited in public databases. (PDF 690 kb)

Additional file 6: **Table S2.** Summary of cDNA sequence reads from each library aligned to the assembly. For each RNA-Seq library (lines), Additional file Table 2 shows the number of sequence reads generated (columns *Left Reads, Right Reads*) and the percentage of theses reads mapping into the transcriptome assembly (columns *Mapped*). (PDF 47 kb)

## Abbreviations

4CL: 4 coumarate-ligase
bp: Base pair
cDNA: Complementary DNA
CHS: Chalcone synthase
DE: Differential expression
GO: Gene ontology
KASS: KEGG automatic annotation server
KEGG: Kyoto encyclopedia of genes and genomes
KO: KEGG orthology
LM: Las Marías
LTP: Lipid transfer
MAL: Major allergen
MET: Metallothionein
ng: Nanograms
OEE: Oxygen evolving enhancer
ORF: Open reading frame
PAL: Phenylalanine ammonia-lyase
PCR: Polymerase chain reaction
PX: Peroxidase
qRT-PCR: Quantitative reverse transcription polymerase chain reaction
s: Seconds
SP: Santo Pipó
TUA: Alpha tubulin
UBQ: Ubiquitin
YM: Yerba mate
