# Supplementary material for: Yerba mate (Ilex paraguariensis, A. St.-Hil.) de novo transcriptome assembly based on tissue specific genomic expression profiles
Source: BMC Genomics. 2018 Dec 7;19:891. doi: 10.1186/s12864-018-5240-6 (PMC6286616; doi:10.1186/s12864-018-5240-6)
Supplement: Supplementary file 1 — Figure S1. Tissue samples and starting material. A: Samples were collected from different tissues to maximise the transcriptional activity coverage: normal leaves, curly leaves, seedling and root. B: Extent of the damaged caused by curly leaves on the tip of a single branch (Ilex paraguariensis) (PDF 2741 kb) [file 12864_2018_5240_MOESM1_ESM.pdf]

Supplementary Figure 1: Tissue samples of *Ilex paraguariensis.*

A

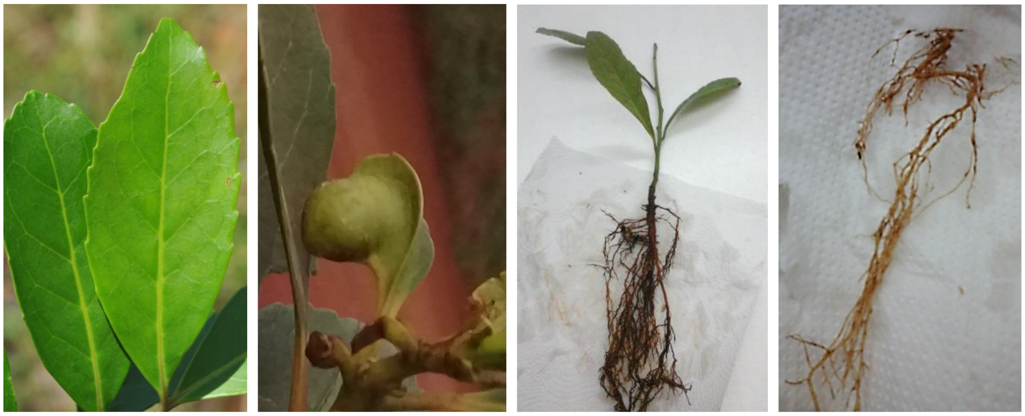

normal leaf	curly leaf	seedling	root

B

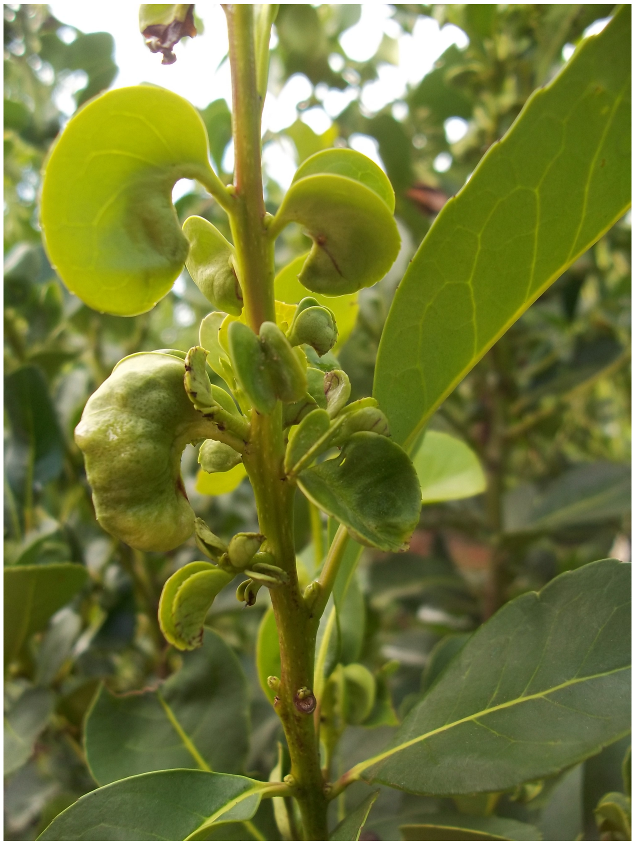

Curly leaf phenotype: Extent of the damaged in a single branch.
