# Supplementary material for: Yerba mate (Ilex paraguariensis, A. St.-Hil.) de novo transcriptome assembly based on tissue specific genomic expression profiles
Source: BMC Genomics. 2018 Dec 7;19:891. doi: 10.1186/s12864-018-5240-6 (PMC6286616; doi:10.1186/s12864-018-5240-6)
Supplement: Supplementary file 5 — Figure S2. Experimental validation of Ilex paraguariensis transcriptome assembly. PCR primers, PCR products and sequence identity of amplified cDNA sequences of 11 predicted genes in the Ilex paraguariensis transcriptome. A: List of 11 genes identified in the transcriptome annotation, the corresponding PCR primers sequence and expected amplicon size. B: PCR products matching expected size: 100, 150 and 1200 bp. C: Amplicon DNA sequence BLASTx results showing significant sequence identity to DNA sequences from other species deposited in public databases. (PDF 690 kb) [file 12864_2018_5240_MOESM5_ESM.pdf]

Supplementary Figure 2

A

	Transcript_id	Description	**Primer** sequence	Amplicon expected size (pb)
**1**	comp103593_c0_seq1	Alpha-tubulin	F_GCCTGATGTTCCGTGGTGAT<br>R_ GTTGGGCACCAGTCGACAA	100
**2**	comp112022_c0_seq17	Ubiquitin	F_AGCCCACACTTGCCACAGTAA<br>R_ AAGTTCAGAGGCTGAGGAAGGA	100
**3**	comp22034_c0_seq1	Plastocyanin	F_GGAAAGCAGCGTTGTTCTTGA<br>R_ GTCTTGCTCGGTGCTGATGA	100
**4**	comp67312_c0_seq1	Metallothionein-like protein type 2	F_TGGTGTTGCACCCCAGAAG<br>R_GCAAGGATCACAGGTGCAGTT	100
**5**	comp108576_c2_seq1	Major allergen	F_GGTGGTGTTGGCAGCATTAA<br>R_AGTGGAACTTGCTTGCATCAAG	100
**6**	comp96468_c0_seq1	Lipid transfer protein	F_GGGATCAGGGCACTCAACAG<br>R_ GGTTGATACCGCTGATGGATTT	100
**7**	comp22041_c0_seq1	Peroxidase	F_GCATAGACACCCCCGGAGTT<br>R_GGATCCACCTCTGGGTACAAAC	100
**8**	comp72426_c0_seq1	Oxygen-evolving enhancer protein 1	F_GCATAGACACCCCCGGAGTT<br>R_ GGATCCACCTCTGGGTACAAAC	100
**9**	comp122133_c0_seq1	Chalcon syntasa	F_GGGTGGGCAATCCAGAAGAT<br>R_AGATGGGCACTTGCGTGAAG	150
**10**	comp122412_c0_seq1	4-coumarate--CoA ligase	F_GCTCTCGTCCCTGCGTGAT<br>R_CCGGCGAGTTCTGGAGTAAA	150
**11**	comp115046_c1_seq1	Phenylalanine ammonia-lyase	F_GGTAGCCACTTGGATGAGGT<br>R_GCACTCTCGACCTCTTTTGG	1200

B

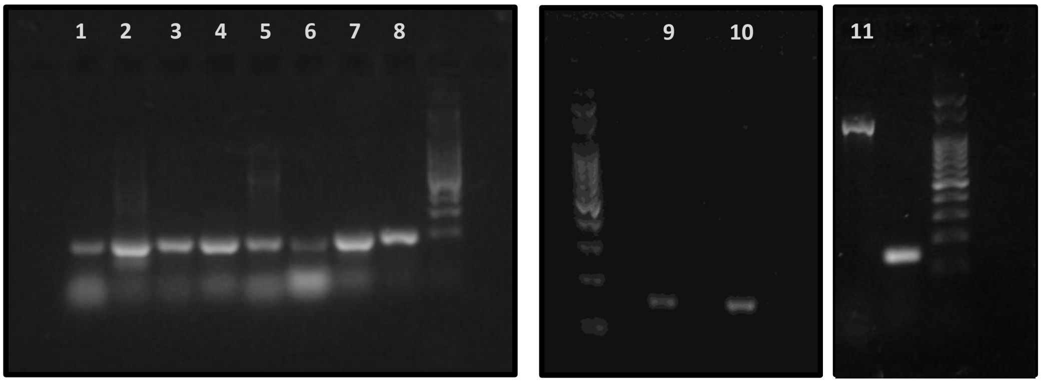

C

	Description	Query cover	E value	Identity	Accession
**1**	Alpha-tubulin [*Populus alba*]	90%	2.4	100%	AFR42124.1
**2**	Ubiquitin [*Gossypium hirsutum*]	91%	0.46	100%	AAO92744.1
**3**	Metallothionin 1 [*Camellia sinensis*]	95%	3.4	100%	ABD97257.1
**4**	Plastocyanin, chloroplastic [*Glycine soja*]	95%	0.00000004	79%	KHN33826.1
**5**	Oxygen-evolving enhancer protein [*Medicago truncatula*]	71%	0.24	88%	XP_003611860.1
**6**	Peroxidase [*Populus tomentosa*]	95%	0.0001	95%	AKE81099.1
**7**	Lipid transfer protein-like [*Arabidopsis thaliana*]	92%	8.8	81%	BAB09777.1
**8**	Major allergen Pru ar 1-like [*Nelumbo nucifera*]	98%	7.2	72%	XP_010245449.1
**9**	4-coumarate CoA ligase [*Lonicera hypoglauca*]	97%	3E-19	78%	AGE10609.1
**10**	Chalcone synthase [*Morus alba* var. multicaulis]	98%	7E-25	98%	AHL83549.1
**11**	Phenylalanine ammonia-lyase [*Perilla frutescens*]	96%	0	89%	AEZ67457.1
