# Supplementary material for: Yerba mate (Ilex paraguariensis, A. St.-Hil.) de novo transcriptome assembly based on tissue specific genomic expression profiles
Source: BMC Genomics. 2018 Dec 7;19:891. doi: 10.1186/s12864-018-5240-6 (PMC6286616; doi:10.1186/s12864-018-5240-6)
Supplement: Supplementary file 6 — Table S2. Summary of cDNA sequence reads from each library aligned to the assembly. For each RNA-Seq library (lines), Additional file Table 2 shows the number of sequence reads generated (columns Left Reads, Right Reads) and the percentage of theses reads mapping into the transcriptome assembly (columns Mapped). (PDF 47 kb) [file 12864_2018_5240_MOESM6_ESM.pdf]

Supplementary Table 2

RNA-Seq Library	***Left Reads***	***Mapped***	***Right_Reads***	***Mapped***
LIB8291	15,195,091	13.401.801 (88.2%)	15,195,091	12.989.035 (85.5%)
LIB8292	15,920,071	12.712.628 (79.9%)	15,920,071	12.353.486 (77.6%)
LIB8293	14,915,294	11.900.530 (79.8%)	14,915,294	11.524.249 (77.3%)
LIB8294	17,969,233	14.161.470 (78.8%)	17,969,233	13.537.025 (75.3%)
LIB8295	15,508,697	11.668.128 (75.2%)	15,508,697	11.313.468 (72.9%)
LIB8296	22,879,049	17.221.764 (75.3%)	22,879,049	16.686.877 (72.9%)
LIB8297	11,782,818	9.922.167 (84.2%)	11,782,818	9.559.593 (81.1%)
LIB8298	8,896,863	7.666.613 (86.2%)	8,896,863	7.394.110 (83.1%)
LIB8299	15,878,954	13.751.737 (86.6%)	15,878,954	13.375.004 (84.2%)
LIB8300	16,444,957	14.264.217 (86.7%)	16,444,957	13.859.514 (84.3%)
LIB8301	25,641,874	22.142.630 (86.4%)	25,641,874	21.432.396 (83.6%)
LIB8302	25,477,268	19.926.243 (78.2%)	25,477,268	19.293.722 (75.7%)
LIB8303	21,861,154	16.916.093 (77.4%)	21,861,154	16.417.969 (75.1%)
LIB8304	22,544,064	19.854.051 (88.1%)	22,544,064	19.158.234 (85.0%)
LIB8305	26,346,560	24.052.300 (91.3%)	26,346,560	23.320.168 (88.5%)
